# Editorial: Biology and Pharmacological Effects of Extracellular Vesicles in Cancer

**DOI:** 10.3389/fmolb.2022.896561

**Published:** 2022-04-25

**Authors:** Jian-ye Zhang, Jiang-Jiang Qin, Dongmei Zhang, Dong-Hua Yang

**Affiliations:** ^1^ Guangzhou Municipal and Guangdong Provincial Key Laboratory of Molecular Target & Clinical Pharmacology, The NMPA and State Key Laboratory of Respiratory Disease, School of Pharmaceutical Sciences and the Fifth Affiliated Hospital, Guangzhou Medical University, Guangzhou, China; ^2^ The Cancer Hospital of the University of Chinese Academy of Sciences (Zhejiang Cancer Hospital), Institute of Basic Medicine and Cancer (IBMC), Chinese Academy of Sciences, Hangzhou, China; ^3^ College of Pharmacy, Jinan University, Guangzhou, China; ^4^ Department of Pharmaceutical Sciences, College of Pharmacy and Health Sciences, St. John’s University, Queens, NY, United States

**Keywords:** extracellular vesicles, contents, biology, pharmacological effects, cancer

The term extracellular vesicles (EVs) generally refers to various nanoscale membrane vesicles secreted by most eukaryotic cells into extracellular environment. EVs include exosomes, microvesicles, and apoptotic bodies. EVs have attracted numerous attention of biomedical investigators and their roles in intercellular communication in multiple physiological and pathological processes have been widely studied. This Research Topic collates the research findings which illustrate the biological and pharmacological roles of EVs in cancer. The topic consists of 14 articles, including 8 review articles and 6 original research articles, contributed by more than 128 authors in the fields of cancer pharmacology and therapeutics. Our goal was to reveal the detailed molecular mechanism of EVs in mediating tumorigenesis and development, and to open new approaches for the clinical therapeutics of cancer.

EVs contain various bioactive molecules, including proteins, lipids, mitochondrial DNA, RNAs and metabolites. Among them, non-coding RNA (ncRNA), especially microRNAs (miRNAs) and long non-coding RNAs (lncRNAs), have been extensively investigated in cancer migration, metastasis, drug resistance, and immunosuppression. Zheng et al. found that gastric cancer-derived exosomal miR-590-5p inhibited gastric cancer cell migration and invasion *in vitro*. In addition, serum exosomal miR-590-5p expression was significantly low in gastric cancer patients, and the expression of miR-590-5p was strongly associated with the TNM stage and the survival rate of gastric cancer patients. A study performed by Xuan et al. showed that exosomal miR-549a derived from tyrosine kinase inhibitors (TKI)-resistant renal cancer possessed a stronger ability to promote vascular permeability, angiogenesis and tumor lung metastasis in nude mice model, compared with sensitive tumor cells. Their mechanistic studies found that exosomal miR-549a regulated the VEGFR2-ERK-XPO5 pathway mainly by activating HIF1α. In line with the article of Xuan’s group, the review of Huang et al. discussed recent reports on tumor-derived EVs and their cargoes, especially ncRNAs and proteins, on tumor angiogenesis and their mechanisms. Xue et al. provided evidence that miR-317b-5b-loaded engineered exosomes could be internalized by tumor cells, subsequently inhibiting cell proliferation, migration, invasion, and inducing cell apoptosis.

In their review articles, Shan et al. discussed the different roles of M1 and M2 subtype macrophages-derived exosomes in tumor progression. M2 macrophages-derived exosomal miRNA could suppress proliferation, migration, invasion, and promote apoptosis of tumor cells, while M1 macrophages-derived exosomal miRNA showed diametrically opposed effects. In addition, Izadirad et al. summarized the roles of EVs-derived miRNAs in the development of acute myeloid leukemia and acute lymphoblastic leukemia, and discussed the prognostic value of EVs in the clinical setting of leukemia. Similarly, Zhang et al. focused on the biology and function of EVs in cancer development. These findings suggest that EVs-derived ncRNAs might become novel strategies for cancer therapy.

EVs, particularly exosomes, are considered to be an excellent drug delivery vehicle due to their membrane-enclosed structure and good biocompatibility. Liu et al. illustrated that adeno-associated virus (AAV)-containing exosomes (AAVExo) apparently improved the gene transfer efficiency in a variety of lung cancer cell types both *in vitro and in vivo* compared to conventional AAV vector. Previous studies have shown that AAV-mediated gene transfer is easily blocked by neutralizing antibodies in human serum when used in the treatment of cancer, resulting in unsatisfactory effects. Therefore, AAVExo may enabled the application of a new exosome-based vector to therapeutic treatments of lung cancers. The review of Song et al. discussed the most recent advances in exosomes as natural product delivery carriers in cancer therapy. They gathered evidence that exosomes loaded with natural products such as paclitaxel, curcumin, doxorubicin, celastrol, and β-elemene showed enhanced anti-tumor efficacy. Consistently, in another review, Shan et al. summarized that macrophages-derived exosomes loaded with paclitaxel or adriamycin showed enhanced anti-tumor effects. Wu et al. reviewed that the use of exosomes to control ferroptosis in targeted cells is promising for cancer therapy. The authors summarized an opinion that ferroptosis-inducing drugs such as erastin and newly recognized natural ferroptosis-inducing compounds could be loaded onto exosomes to provide new strategies and approaches for tumor therapy.

Crosstalk between EVs and the tumor microenvironment has important implications for cancer migration and metastasis. The review of Dong et al. highlighted that EVs was critical for the formation of tumor pre-metastatic niche. Tumor-derived EVs promoted tumor cells colonization in distant organs through increasing vascular permeability, extracellular matrix remodeling, angiogenesis and immunosuppression. Similarly, Bao et al. also discussed the biological functions of tumor-derived EVs in reprograming tumor microenvironment.

Interestingly, Sadovska et al. found that exercise-induced EVs could delay the progression of prostate cancer. RNA-sequencing analysis showed substantial changes in the RNA content of EVs collected before and after exercise in rats. Exercise-induced EVs significantly inhibit lung metastasis of prostate cancer cells in rats. Therefore, the research supported the idea that regular physical exercise should be prescribed to prostate cancer patients as a tertiary prevention measure. Bandini et al. focused on potentially useful biomarkers in breast cancer-derived EVs for diagnosis and monitoring. They identified 11 biomarkers in plasma EVs that could be used to significantly distinguish healthy subjects from breast cancer patients, including CD3, CD56, CD2, CD25, CD9, CD44, CD326, CD133/1, CD142, CD45, and CD14.

In summary, the Research Topic of “Biology and Pharmacological Effects of Extracellular Vesicles in Cancer” highlights the important roles of EVs in regulating tumor proliferation, migration, metastasis, immune escape, inflammatory response and drug resistance. With the continuous in-depth study on the mechanism by which EVs regulate the biological behavior of tumors, EVs-based therapy will become a new avenue of cancer treatment.

